# Survey on the Awareness of Menstruation and Menstruation-Related Problems in Athletes among Public High School Physical Education Teachers

**DOI:** 10.31662/jmaj.2022-0054

**Published:** 2022-12-12

**Authors:** Anna Takabayashi-Ebina, Tsuyoshi Higuchi, Minako Yokoyama, Maika Oishi, Yoshihito Yokoyama

**Affiliations:** 1 Department of Obstetrics and Gynecology, Hirosaki University, Graduate School of Medicine, Hirosaki, Japan; 2 Department of Nursing Science, Hirosaki University, Graduate School of Health Sciences, Hirosaki, Japan

**Keywords:** female athletes, high school teachers, menstruation, menstrual problems

## Abstract

**Introduction:**

In recent years, the activities of female athletes have attracted increasing attention, especially regarding the effect of menstruation on athletic performance. Nevertheless, there are no surveys of these practices among coaches who train non-top-level athletes for general competition. This study aimed to investigate how high school physical education teachers approach the issue of menstruation and the awareness of menstruation-related problems.

**Methods:**

This was a questionnaire-based cross-sectional study. The participants were 225 health and physical education teachers from 50 public high schools in the Aomori Prefecture. Participants were asked to answer a questionnaire regarding whether they talk to their female athletes about menstruation, keep track of their menstrual status, or make adjustments for menstruating students. Additionally, we asked for their views on painkiller use and their knowledge of menstruation.

**Results:**

The participants included 183 men (81.3%) and 42 women (18.7%); data from 221 participants were analyzed after four teachers were excluded. Teachers of female athletes who communicated with students regarding their menstrual conditions and physical changes were predominantly female (p < 0.01). Regarding the use of painkillers for menstrual pain, more than 70% of respondents said that they recommended their active use. Few respondents reported that they would adjust a game because of athletes with menstrual problems. More than 90% of the respondents knew that there was a change in performance due to the menstrual cycle, and 57% of the respondents understood the relationship between amenorrhea and osteoporosis.

**Conclusions:**

Menstruation-related problems are not only issues for top athletes but also important for general competition level athletes. Hence, even in high school clubs, teachers should be educated on how to deal with menstruation-related problems to prevent withdrawal from sports, maximize athletes’ abilities, prevent future diseases, and preserve fertility.

## Introduction

In 2021, Japan hosted the Olympic Games, and 14 of the 27 gold medals awarded to Japanese athletes were won by women. Conversely, female athletes, similar to women in general, experience menstrual problems, such as menstrual pain, dysmenorrhea, abnormal menstrual cycles, and premenstrual syndrome (PMS)/premenstrual dysphoric disorder (PMDD). PMS is a mental or physical symptom that lasts for 3-10 days before menstruation, and it alleviates or disappears with the onset of menstruation. PMDD refers to the symptoms of PMS that are mainly mental in origin. Evidently, there is a need for competition instructions specific to the characteristics of female athletes.

Sports-related menstrual problems include changes in conditions due to the menstrual cycle, weight loss amenorrhea, and the risk of osteoporosis due to long-term amenorrhea ^(1)^. Recent research has focused on the female athlete triad (FAT), which is defined as a combination of low energy availability, menstrual dysfunction, and low bone mineral density ^(2), (3)^. Maintaining the condition of female athletes to maximize their competitive ability is directly associated with competition results, and previous research has reported that the physical and mental conditions of female athletes are affected by hormones, whose levels fluctuate throughout the menstrual cycle ^(4)^. There are several ways to cope with the changes in physical conditions caused by menstruation, such as controlling menstruation pain through analgesics, that is, relieving abdominal pain, back pain, and headaches during menstruation, and via “menstrual movements,” that is, shifting menstrual periods to avoid any coincidence with the schedule of games. Nevertheless, these methods are not widely used in Japan. Previous reports have shown that the premenstrual phase is associated with worse performance and an increased incidence of injury ^(5), (6)^. Furthermore, PMS and PMDD are common menstrual problems in female collegiate athletes, and almost 50% of athletes have reported experiencing the negative effect of premenstrual symptoms on athletic performance ^(7), (8)^. Given that many sports coaches are men, it is important to ensure that knowledge and information related to gynecology and menstruation are sufficiently communicated to maintain the health of female athletes. However, FAT is not just a problem for athletes who undergo intense training; it can also occur at the school sports level ^(9)^.

A systematic review reported that the prevalence of all three components of the FAT in high school girls, college students, and elite athletes ranged from 0% to 16%; the prevalence of two components ranged from 3% to 27%, and the prevalence of a single component ranged from 16% to 60% ^(10)^. This shows that this issue is important in nonprofessional female athletes.

Many surveys have focused on how sports coaches relate to athletes concerning menstruation and how instructors of top-level athletes adjust their practices to consider menstruation ^(11)^, but there have been no surveys of these practices among coaches who train athletes for general competition. In Japan, teachers in charge of club activities at high schools are not specialized instructors, and their degree of awareness of menstruation-related problems is unknown. Thus, this study aimed to investigate teachers’ perceptions of menstruation and menstruation-related problems according to their sex.

## Materials and Methods

### Ethics

This study was conducted following the Declaration of Helsinki and was approved by the Hirosaki University Graduate school of Medicine Ethics Committee (Approval code: 2019-1104). All of the participants provided informed consent.

### Study population

We invited 291 physical education teachers at 56 public high schools in Aomori Prefecture to complete a questionnaire survey and received responses from 225 participants in 50 schools. The survey response rate was 77.3%. After excluding those who were not in charge of club activities and those in charge of club activities involving music or art, data from 221 participants were analyzed. The participants were divided into three groups based on their and their students’ sex.

### Questionnaire

The questionnaire asked physical education teachers whether they talked to their female athletes about menstruation, kept track of their menstrual status, and made adjustments to compensate for menstruation. The questionnaire also ascertained their knowledge of menstruation and their views on athletes’ use of painkillers. Table 1 lists the items in the questionnaire. For questions 5 and 6, we included an open-ended field for reasons.

**Table 1.** List of Items Used in the Questionnaire Survey.

*Questionnaire*	*Answer*
**Information on respondents**	
Sex	Male/Female
Age	Years old
Club activities you are leading	Male/Female, Free to write
Coaching history	Years
Athletic experience in the club you are coaching	Yes/No
Other athletic experience	Yes/No
**Questions**
Q1: Have you ever talked about menstrual status or physical condition?	Yes/No
Q2: Do you understand the athlete's menstrual cycle and menstrual symptoms?	Yes/No
Q3: Is it used for practice planning and conditioning?	Yes/No
Q4: What do you do if a player complains of poor health?	Reduce practice/Another menu/Let the practice rest/ Let the person choose
Q5: What are your thoughts on the use of analgesics for menstrual cramps?	Actively use/Better to use/Don’t use/Other
	Free description of the reason
Q6: What are your thoughts on athletes with menstrual problems moving their menstruation for games?	Actively use/ Unavoidable/Don’t use
	Free description of the reason
Q7: Do you know that the menstrual cycle changes your physical condition and performance?	Know/Do not know
Q8: Do you know about premenstrual syndrome, a condition that makes you feel sick during the period before the start of menstruation?	Know/Do not know
Q9: Do you know that weight loss can lead to amenorrhea?	Know/Do not know
Q10: Do you know that menstruation stopped for a long period of time is a problem?	Know/Do not know
Q11: Do you know that there is a relationship between stopped menstruation and osteoporosis?	Know/Do not know
Q12: Do you know what is the Female Athlete Triad?	Know/Do not know

Regarding the use of painkillers for menstrual pain and adjustment for menstruation among athletes with menstrual problems, the questionnaire was scored as follows: one point for not recommending their use, two points for leaving this decision to the student, three points for encouraging their use, and four points for strongly recommending their use.

### Statistical analyses

Statistical analyses were performed using SPSS version 27 (IBM, Armonk, NY, USA) and R version 4.0.2 (R Foundation for Statistical Computing, Vienna, Austria). Analysis of variance was used to compare the responses to the questions between the three groups. Statistical significance was set at P < 0.05.

## Results

**Figure 1.**
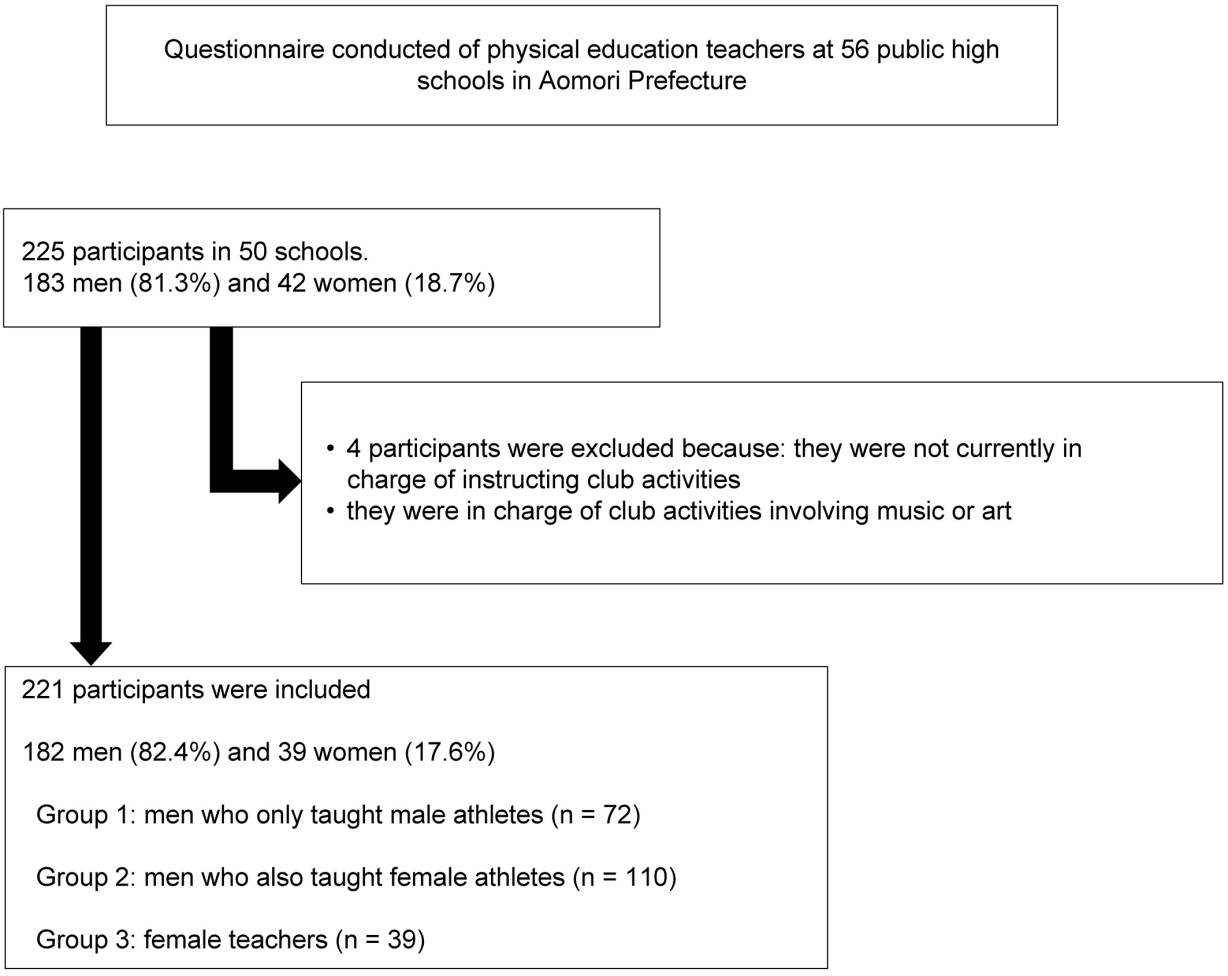
Flowchart of the questionnaire administration and application of the exclusion criteria.

Table 2 summarizes the characteristics of the study population. The respondents included 183 men (81.3%) and 42 women (18.7%). After excluding those who were not currently in charge of instructing club activities and those in charge of club activities involving music or art, data from 221 participants were analyzed (Figure 1). The teachers were divided into three groups as follows: men who only taught male athletes (Group 1, n = 72), men who also taught female athletes (Group 2, n = 110), and women (Group 3, n = 39). Among the female teachers surveyed, their student groups were either coeducational or female-only, that is, no female teachers taught groups of only male students. The overall mean age was 40.6 (standard deviation [SD] 11.0) years; age was significantly different among the three groups. The mean duration of teaching experience was 12.9 (SD 11.1) years; teaching experience was also significantly different among the three groups. Multiple comparisons showed significant differences between Groups 2 and 3 in both cases, indicating that the younger age of Group 3 also resulted in fewer years of instruction.

**Table 2.** Characteristics of Study Participants (n = 221).

**Characteristics**	Total sample (n = 221)	Group 1 (n = 72)	Group 2 (n = 110)	Group 3 (n =39 )	p-value
Age (years), mean±SD	40.6 ± 11.0	39.8 ± 10.0	42.5 ± 11.5	37.1 ± 11.0	0.02
Years of instruction, mean±SD	12.9 ± 11.1	13.0 ± 10.1	14.5 ± 12.1	8.1 ± 8.6	0.002
**Type of sport, n (%)**					
Athletics	34 (15.4)	1 (1.4)	28 (25.5)	5 (12.8)	
Baseball	31 (14.0)	31 (43.1)	0 (0)	0 (0)	
Basketball	26 (11.8)	6 (8.3)	12 (10.9)	8 (20.5)	
Kendo	17 (7.7)	1 (1.4)	16 (14.5)	0 (0)	
Volleyball	16 (7.2)	4 (5.5)	7 (6.4)	5 (12.8)	
Badminton	12 (5.4)	1 (1.4)	7 (6.4)	4 (10.3)	
Football	11 (5.0)	10 (13.9)	1 (0.9)	0 (0)	
Others	74 (33.5)	18 (25.0)	39 (35.4)	17 (43.6)	

Group 1: men who only taught male athletes (n = 72), Group 2: men who taught male and female athletes (n = 110), and Group 3: female teachers (n = 39). SD: standard deviation

Table 3 shows the questionnaire items and teacher responses to questions regarding the awareness of menstruation-related issues. For question 4, the number of responses in Group 2 was larger than the sample size because some respondents chose more than one option. Teachers who were in charge of female athletes and communicated effectively with athletes about their menstrual conditions and changes in their physical condition were predominantly women (p = 0.000035). Nevertheless, few teachers fully understood issues related to the menstrual cycle, and most responded that they deduced the menstrual conditions and changes in physical condition from the physical state of the athlete or did not know about the menstrual conditions and changes in the physical condition of the athletes. Furthermore, even if the menstrual cycle and changes in physical condition were understood, the ratio of using menstrual cycle information for practice planning and adjustment was not high (25%).

**Table 3.** Response to Athletes’ Menstruation.

**Q1: Have you ever talked about menstrual status or physical condition? n (%)**			
	Group 2 (n = 110)	Group 3 (n = 39)	
Yes	68 (61.8)	37 (94.9)	p = 0.000035
No	42 (38.2)	2 (5.1)	
No response	0 (0)	0 (0)	
**Q2: Do you understand the athlete's menstrual cycle and menstrual symptoms? n (%)**			
	Group 2 (n = 110)	Group 3 (n = 39)	
Grasp everyone	0 (0)	0 (0)	p = 0.041
Partially grasped	26 (23.6)	10 (25.7)	
Inferred from the state of the players	48 (43.7)	24 (61.5)	
Do not know	36 (32.7)	5 (12.8)	
No response	0 (0)	0 (0)	
**Q3: Is it used for practice planning and conditioning? n (%)**			
	Group 2 (n = 110)	Group 3 (n = 39)	
Yes	26 (23.6)	8 (20.5)	p = 0.82
No	58 (52.7)	21 (53.9)	
No response	26 (23.6)	10 (25.6)	
Q4: What do you do if a player complains of poor health? n (%)*			
	Group 2 (n = 117)	Group 3 (n = 42)	
Reduce practice	5 (4.2)	2 (4.7)	p = 0.32
Another menu	15 (12.5)	3 (7.0)	
Let the person rest	52 (43.3)	15 (34.9)	
Let the person choose	45 (37.5)	22 (51.1)	
No response	0 (0)	0 (0)	

Group 1: men who only taught male athletes, Group 2: men who taught male and female athletes, and Group 3: female teachers Values are presented as n (%). Q1-Q6: Fisher’s exact test *The total is larger than the sample size of this study because some respondents chose more than one option for Q4.

Table 4 shows the teachers’ knowledge of menstruation-related problems. Regarding the use of painkillers for menstrual pain, more than 70% of the respondents in all groups stated that they recommended their active use. A comparison of the weighted averages revealed no significant difference between teachers in recommending the use of painkillers for menstrual pain; however, female teachers tended to have a higher score than male teachers in this respect, and female teachers tended to be more positive about the use of analgesics (p = 0.054). Regarding the adjustments made to prepare menstruating athletes with menstrual problems for competitions, few respondents positively recommended menstrual movement, and no significant difference was noted among the groups. For the questions about the use of analgesics and menstrual movements, the reasons given for the answers selected were positive: eliminating the pain is better and shifting menstruation to improve conditions is a good method, whereas negative opinions stated that such interventions are not good for the body and natural is better and that they had concerns about doping.

**Table 4.** Menstruation-Related Knowledge.

**Q5: About the use of painkillers n (%)**				
	Group 1 (n = 72)	Group 2 (n = 110)	Group 3 (n = 39)	
Actively use	5 (6.9)	7 (6.4)	7 (18.0)	p = 0.17
Better to use	49 (68.1)	72 (65.5)	27 (69.2)	
Don’t use	4 (5.6)	2 (1.8)	0 (0)	
Other	11 (15.3)	26 (23.6)	5 (12.8)	
No response	3 (4.1)	3 (2.7)	0 (0)	
**Q6: About menstrual movement n (%)**				
	Group 1 (n = 72)	Group 2 (n = 110)	Group 3 (n = 39)	
Actively use	9 (12.5)	22 (20.0)	5 (12.8)	p = 0.28
Unavoidable	55 (76.4)	75 (68.2)	33 (84.6)	
Don’t use	7 (9.7)	12 (10.9)	1 (2.6)	
No response	1 (1.4)	1 (0.9)	0 (0)
**Q7: Changes in physical condition due to the menstrual cycle n (%)**				
	Group 1 (n = 72)	Group 2 (n = 110)	Group 3 (n = 39)	
Know	63 (87.5)	101 (91.8)	39 (100)	p = 0.081
Not known	8 (11.1)	9 (8.2)	0 (0)	
No response	1 (1.4)	0 (0)	0 (0)	
**Q8: Premenstrual syndrome n (%)**				
	Group 1 (n = 72)	Group 2 (n = 110)	Group 3 (n = 39)	
Known	48 (66.7)	77 (70.0)	36 (92.3)	p = 0.0074
Not known	22 (30.5)	33 (30.0)	3 (7.7)	
No response	2 (2.8)	0 (0)	0 (0)	
**Q9: Weight loss amenorrhea n (%)**				
	Group 1 (n = 72)	Group 2 (n = 110)	Group 3 (n = 39)	
Know	55 (76.4)	91 (82.7)	37 (94.9)	p = 0.052
Not known	16 (22.2)	37 (94.9)	2 (5.1)	
No response	1 (1.4)	0 (0)	0 (0)	
**Q10: Long-term amenorrhea is a problem n (%)**				
	Group 1 (n = 72)	Group 2 (n = 110)	Group 3 (n = 39)	
Know	69 (95.8)	108 (98.2)	39 (100)	p = 0.66
Not known	2 (2.8)	2 (1.8)	0 (0)	
No response	1 (1.4)	0 (0)	0 (0)	
**Q11: Relationship between amenorrhea and osteoporosis n (%)**				
	Group 1 (n = 72)	Group 2 (n = 110)	Group 3 (n = 39)	
Know	33 (45.8)	64 (58.2)	28 (71.8)	p = 0.034
Not known	38 (52.8)	46 (41.8)	11 (28.2)	
No response	1 (1.4)	0 (0)	0 (0)	
**Q12: About female athlete triad n (%)**
	Group 1 (n = 72)	Group 2 (n = 110)	Group 3 (n = 39)	
Know	9 (12.5)	27 (24.5)	17 (43.6)	p = 0.0017
Not known	59 (81.9)	82 (74.6)	21 (53.8)	
No response	4 (5.6)	1 (0.9)	1 (2.6)	

Group 1: men who only taught male athletes, Group 2: men who taught male and female athletes, and Group 3: female teachers Values are presented as n (%) Q5-Q13: Fisher’s exact test

More than 90% of the respondents understood that performance changes occurred because of the menstrual cycle. Significantly fewer men knew about PMS (p = 0.0074) and weight loss amenorrhea (p = 0.052) compared with women. There was no significant difference between men and women in their knowledge of long-term amenorrhea, with 98% of the respondents being aware of this issue. Additionally, 57% of the respondents were aware of the relationship between amenorrhea and osteoporosis, and 43% were not aware of such a relationship. Finally, only 24% of the respondents knew about FAT, and this predominantly comprised female teachers (p = 0.0017).

## Discussion

Many athletes are aware of changes in performance due to the menstrual cycle ^(4), (5), (7), (9), (12), (13)^. In a previous survey of top-level Japanese athletes, 70% reported experiencing PMS ^(12)^. Additionally, previous studies have reported that approximately 40% of high school and college athletes are aware of poor performance due to PMS ^(5), (7)^. We found that female teachers knew more about menstrual problems and menstruation-related symptoms than their male counterparts. Furthermore, although more than 90% of respondents understood that the menstrual cycle could affect performance, significantly fewer men knew about PMS than women.

The teachers instructing female athletes, who communicated with athletes about their menstrual conditions and changes in their physical condition, were predominantly female; however, few teachers actually used this knowledge to introduce changes to the training program. This could be because only one or a few teachers were responsible for the instruction of many athletes, making it difficult to appraise students individually.

There was no significant between-group difference in recommending the use of analgesics during menstruation, and >70% of the respondents said that they would recommend their active use in the groups. Regarding the use of analgesics during menstruation, on comparing the weighted averages, we found that female teachers tended to recommend more active analgesic use. However, menstrual movement was rarely recommended in the groups, and there were many cautious opinions regarding it.

In other countries, as of 2008, 83% of athletes reported using oral contraceptives ^(14)^. Among Japanese athletes, 7% of athletes who participated in the 2012 London Olympics reported using an oral contraceptive, and this increased to 27.4% of athletes who participated in the 2016 Rio de Janeiro Olympics ^(11)^.

Oral contraceptives have been widely used in the United States since the 1960s. In Japan, oral contraceptives were approved in 1999 and ratified as a treatment for dysmenorrhea in 2008; however, they are not widely used according to the responses to this survey. The reasons for dissuading athletes from taking analgesics or menstrual regulation drugs to adjust the timing to accommodate upcoming competitions included nonscientific reasons such as they are “not good for the body” and “natural is better,” which seem to indicate false perceptions and doping concerns among sports coaches. Sports instructors should have correct knowledge about analgesics and oral contraceptives; indeed, an increasing number of studies have reported that oral contraceptives do not alter aerobic exercise capacity ^(15), (16)^, and they are not prohibited substances.

Among the problems related to menstruation, awareness of the relationship between amenorrhea and osteoporosis was particularly low. High levels of stress, such as overtraining or rapid weight loss; increased cortisol levels; and reduced production of hormones important for menstruation, including gonadotropin-releasing hormone, follicle-stimulating hormone, luteinizing hormone, and estrogen ^(17)^ can impair the regulation of sex hormone secretion in the hypothalamus, leading to amenorrhea and even osteoporosis ^(18)^. There have also been reports of worsened athletic performance in athletes with abnormal menstruation compared with the normal menstruation group ^(19), (20)^. Maintaining a normal menstrual cycle during training can lead to more efficient performance ^(19), (20)^. Coaches must be aware of the relationship between these menstrual abnormalities and their effects on training, as well as the relationship between amenorrhea and osteoporosis.

Additionally, although more female teachers knew about FAT than male teachers, less than half of the respondents knew about FAT. The three components of FAT interact with each other, with energy availability directly affecting menstrual status, and energy availability and menstrual status directly affecting bone health ^(3)^. FAT is not just a problem for athletes who undergo intense training; it can also occur in school-level sports ^(9)^. Therefore, high school sports instructors must be aware of FAT.

More than 80% of the respondents were men, which suggests that many sports instructors are men. Additionally, the main job of high school teachers is education centered on lessons, and the importance of club activity guidance in the overall work of high school teachers may not be notably high. In this study, we conducted a survey of physical education teachers, but teachers of other subjects also provided guidance on club activities, and their understanding of menstruation is expected to be lower. Nevertheless, in Japan, most club activities in junior high schools and high schools are taught by teachers rather than specialized coaches.

The Ministry of Education, Culture, Sports, Science and Technology (MEXT) of Japan reported that the percentage of sports instructors, excluding school teachers, who led school club activities in athletic departments in 2016 was 25% in junior high schools and 11% in high schools. The percentage of teachers who were not health and physical education teachers but taught competitive sports despite having no experience was 45.9% in junior high schools and 40.9% in high schools.

In this survey, we found that knowledge of menstruation among teachers who led club activities was low. One reason coaches have little knowledge of menstruation could be because they are nonspecialist sports education teachers. Recruiting sports instructors, not school teachers, to lead school club activities can reduce the burden of sports education on nonspecialist teachers, who are often unable to provide proper technical guidance. The MEXT of Japan reported that the reasons for not employing external sports instructors included the lack of rules and regulations and appropriate personnel, the inability to hold outside sports instructors accountable for accidents during activities, and budget constraints ^(21)^. The resolution of these issues will take time, and the issues raised in our study should be understood by faculty members who are already teachers at educational establishments.

This survey was limited to one prefecture in a regional city in Japan and targeted only high school teachers in charge of health and physical education; thus, a broader survey is needed to confirm the perceptions of a wider range of sports instructors.

The results of this survey were shared with the participants along with the correct information related to menstruation. Additionally, we plan to give feedback at a lecture for high school physical education teachers and school nurses to increase knowledge regarding this issue.

Menstruation-related problems are not only experienced by top athletes but also by general competition level athletes ^(9)^. Therefore, even in high school clubs, instructors should be educated on how to deal with menstruation-related problems to prevent withdrawal from sports activities, maximize athletes’ abilities, prevent future health problems, and preserve fertility.

## Article Information

### Conflicts of Interest

None

### Sources of Funding

This work was supported by a grant (Approval code: 2019-1104) from the Aomori Society of Sports Medicine.

### Acknowledgement

The authors are grateful to everyone at the Department of Obstetrics and Gynecology, Hirosaki University, for their guidance and encouragement. We thank all the members of the Gynecologic Oncology Group of Hirosaki Graduate School of Medicine for their helpful discussions and advice concerning this work.

### Author Contributions

Professor Y. Yokoyama and Dr. M. Yokoyama were involved in the study design and data interpretation. Dr. Higuchi and Dr. Oishi were involved in the data analysis. All authors critically revised the report, commented on the drafts of the manuscript, and approved the final report.

### Approval by Institutional Review Board (IRB)

2019-1104, Hirosaki University Graduate School of Medicine ethics committee.
